# Supplementary material for: A novel immunofluorescent test system for SARS-CoV-2 detection in infected cells
Source: PLoS One. 2024 May 31;19(5):e0304534. doi: 10.1371/journal.pone.0304534 (PMC11142482; doi:10.1371/journal.pone.0304534)
Supplement: S1 File — The file includes the original uncropped and unadjusted images underlying all blot or gel results reported in the figures. (PDF) [file pone.0304534.s001.pdf]

Raw image for Figure 1C Coomassie brilliant blue R250

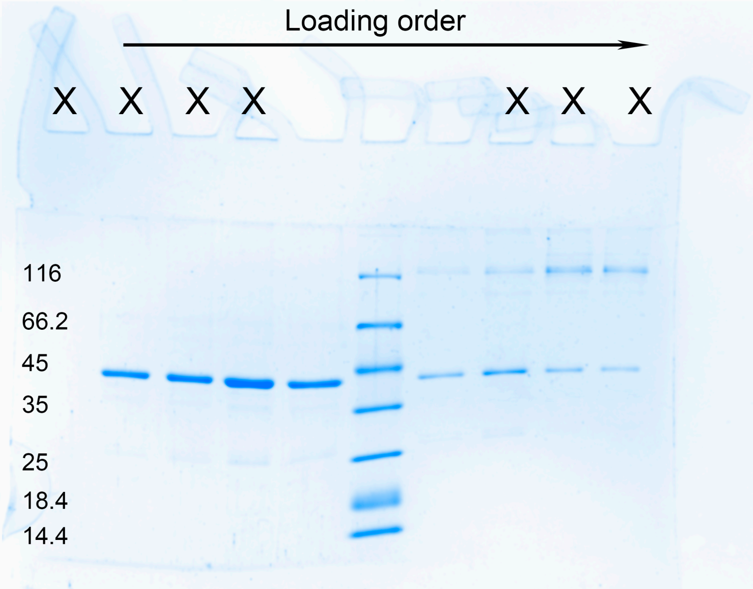

Image obtained by GelDoc EZ Imager

Raw image for Figure 1D DAB staining

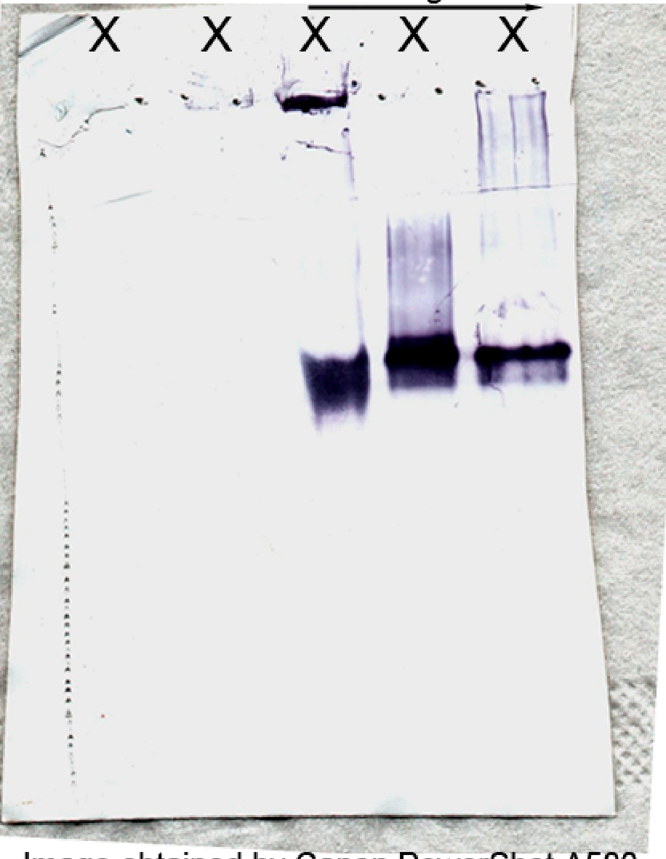

Image obtained by Canon PowerShot A580

Raw image for Figure 1E

UV Detection

Loading order

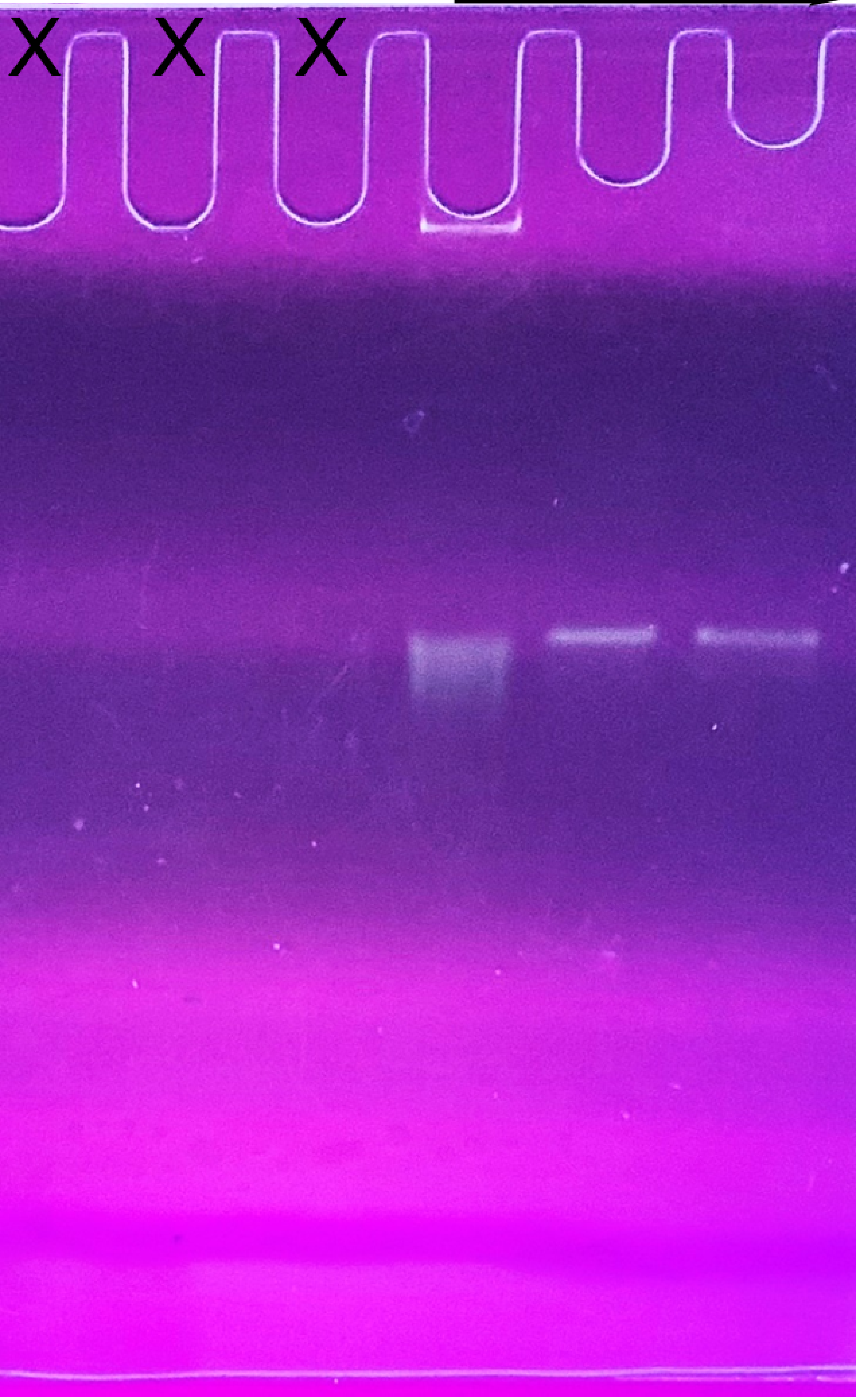

Image obtained by GelDoc EZ Imager

Raw image for Figure 1F

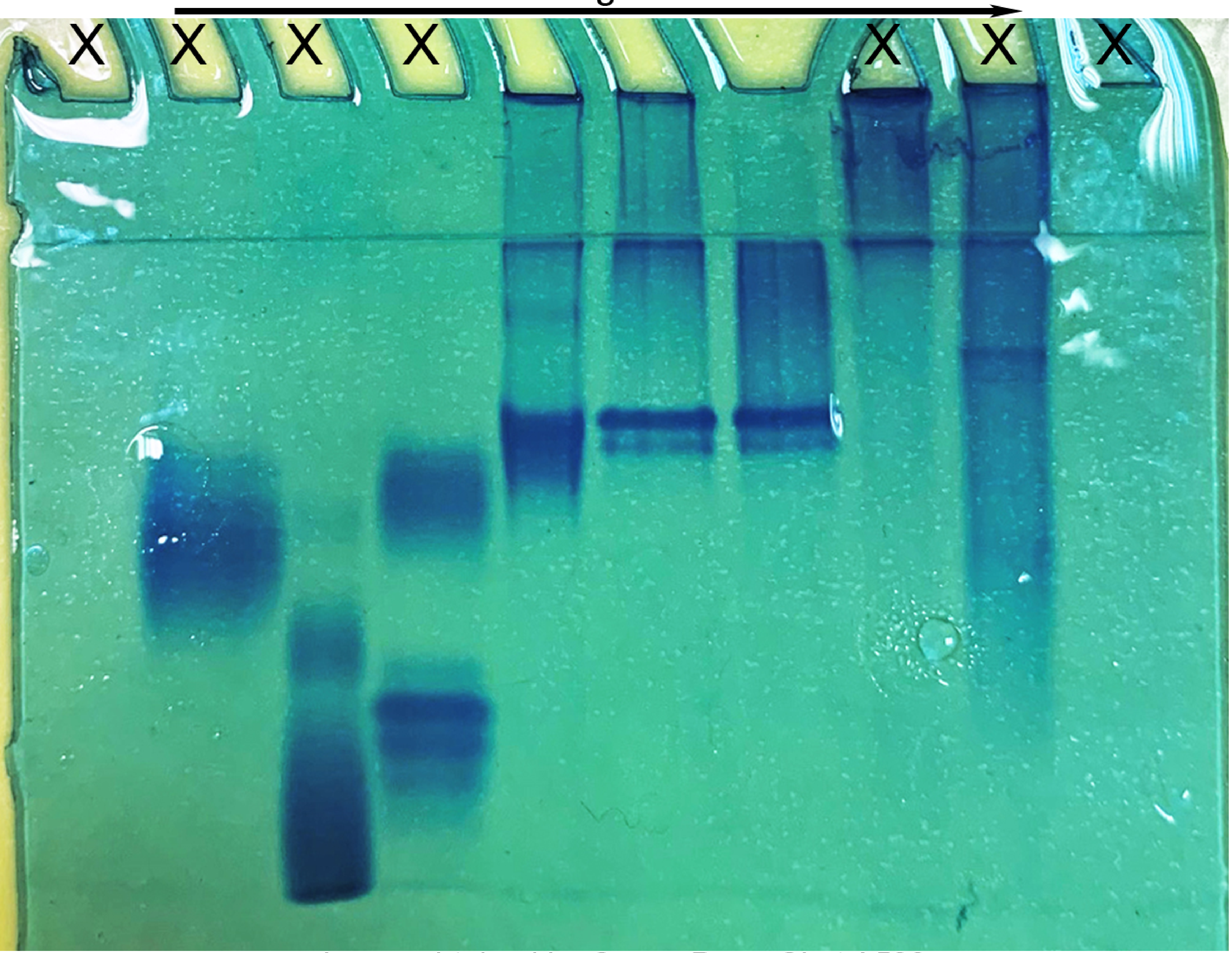

Image obtained by Canon PowerShot A580
