# Supplementary material for: A novel immunofluorescent test system for SARS-CoV-2 detection in infected cells
Source: PLoS One. 2024 May 31;19(5):e0304534. doi: 10.1371/journal.pone.0304534 (PMC11142482; doi:10.1371/journal.pone.0304534)
Supplement: Attachment — Submitted filename: Response to Reviewers.docx [file pone.0304534.s002.docx]

Thank you for consideration of our manuscript entitled "*A novel immunofluorescent test system for SARS-CoV-2 detection in infected cells*”. We appreciate the time and effort dedicated by the reviewer to provide insightful feedback on ways to strengthen our paper. Thus, it is with great pleasure that we resubmit for further consideration our article containing the changes that reflect the suggestions provided. We hope that our edits and responses which are provided below satisfactorily address all the issues and concerns the reviewer has noted.

To facilitate your review of our revisions, the following is a point-by-point response to the referee’s questions and comments.

Comments from Referee:

Reviewer #1: This publication proposes the development of a new analytical tool to detect SARS-CoV-2 by a two-component test system with high sensitivity, a reduced number of analytical steps and low cost.

The manuscript is well written and understandable.

***Authors’ response:*** *Thank you very much for a such positive feedback.*

\# minor recomendations:

The population of vaccinated candidates could be extended to people vaccinated with other vaccines as Astra Zeneca, BioNtech or Moderna. L100: "Thirty serum samples were collected during the period from 2 December 2020 to 12 December 2023 from five subjects fully vaccinated with Sputnik V and twenty-three COVID-19 convalescents aged 31 to 84 years, who participated in the study of humoral and T-cell responses to SARS-CoV-2." For statistical reasons, the number of patients should be greater to validate the study.

***Authors’ response:*** *We thank the reviewer for this important note. We agree with the reviewer regarding the comment about the small sample size of the subjects. However, due to the very limited availability of imported COVID-19 vaccines in our country, we were unable to add serum samples from such vaccinated individuals to the screening. Therefore, we conducted additional studies of neutralizing activity of sera from individuals vaccinated with Sputnik V vaccine (35 samples) and from individuals who had recovered from COVID-19 caused by SARS-CoV-2 (B.1) (36 patients). New results were also obtained side-by-side using the two test systems and a correlation was found for the calculated IC50 values as well as for the previously screened samples. Given these results, the expanded experimental sample included serum samples from 59 COVID-19 convalescents and 40 vaccinated individuals.*

Figure 1 could be improved with particular annotations on Westerblot (figure1C,D,E) for ease of reading.

***Authors’ response:*** *We thank the reviewer for noting this. The figure was corrected accordingly. To better illustrate the agreement between the theoretical and observed mass of the streptavidin-eGFP protein, we added SDS-PAGE with a protein molecular weight marker to Figure 1 (panel C). In addition, in Figure 1, D ,E, F, bands corresponding to the active form of EGFP-SA and incorrectly folded aggregates formed during inadequate storage were indicated.*

Figure2 : the letters and numbers are not sufficiently legible on the tables (modification of size of letters for example). An explanation on negative and positive controls is to be added (in the text or on figures).

***Authors’ response:*** *We thank the reviewer for this critique. Unfortunately, we cannot increase the size of well letters and the numbers indicating the number of FFUs in the resulting images obtained with the vSpot Spectrum Reader. Nevertheless, we added a description of the controls to the corresponding paragraph of the Materials and Methods chapter on performing the microneutralization assay.*

Journal requirements:

When submitting your revision, we need you to address these additional requirements.

1. Please ensure that your manuscript meets PLOS ONE's style requirements, including those for file naming. The PLOS ONE style templates can be found at

<https://journals.plos.org/plosone/s/file?id=wjVg/PLOSOne_formatting_sample_main_body.pdf> and

<https://journals.plos.org/plosone/s/file?id=ba62/PLOSOne_formatting_sample_title_authors_affiliations.pdf>

***Authors’ response:*** *We checked the manuscript for the PLOS ONE's style requirements.*

2. We noticed you have some minor occurrence of overlapping text with the following previous publication(s), which needs to be addressed:

<https://pdfs.semanticscholar.org/755b/684fa2e81cfa38957ed82f7fb90ee6d88405.pdf>

<https://www.mdpi.com/2076-393X/11/12/1810>

***Authors’ response:*** *We revised our manuscript to reduce similarities with our previous publications.*

3. Thank you for stating the following financial disclosure:

“This research was funded by the RSCF grant 21-75-30003.”

Please state what role the funders took in the study.  If the funders had no role, please state: "The funders had no role in study design, data collection and analysis, decision to publish, or preparation of the manuscript."

If this statement is not correct you must amend it as needed.

Please include this amended Role of Funder statement in your cover letter; we will change the online submission form on your behalf.

***Authors’ response:*** *We included the following statement to the manuscript: «The funder had no role in study design, data collection and analysis, decision to publish, or preparation of the manuscript.”.*

4. We note that your Data Availability Statement is currently as follows: [All relevant data are within the manuscript and its Supporting Information files.]

Please confirm at this time whether or not your submission contains all raw data required to replicate the results of your study. Authors must share the “minimal data set” for their submission.

For example, authors should submit the following data:

- The values behind the means, standard deviations and other measures reported;

- The values used to build graphs;

- The points extracted from images for analysis.

Authors do not need to submit their entire data set if only a portion of the data was used in the reported study.

If your submission does not contain these data, please either upload them as Supporting Information files or deposit them to a stable, public repository and provide us with the relevant URLs, DOIs, or accession numbers.

***Authors’ response:*** *We changed the Data Availability Statement for «Data are available from corresponding author who may be contacted by email».*

5. Your ethics statement should only appear in the Methods section of your manuscript. If your ethics statement is written in any section besides the Methods, please delete it from any other section.

***Authors’ response:*** *This statement is now only in the Methods section.*

6. PLOS ONE now requires that authors provide the original uncropped and unadjusted images underlying all blot or gel results reported in a submission’s figures or Supporting Information files.

***Authors’ response:*** *We included raw blot photos to the submission.*

7. Please review your reference list to ensure that it is complete and correct.

***Authors’ response:*** *The list of references was revised and corrected.*
